# Comparison of *Ophiopogon japonicus* and *Liriope spicata* var. *prolifera* from Different Origins Based on Multi-Component Quantification and Anticancer Activity

**DOI:** 10.3390/molecules28031045

**Published:** 2023-01-20

**Authors:** Min-Hui Chen, Fong Leong, Si-Jia Gao, Xin Chen, Jin-Jian Lu, Li-Gen Lin, Yitao Wang, Xiao-Jia Chen

**Affiliations:** 1 State Key Laboratory of Quality Research in Chinese Medicine, Institute of Chinese Medical Sciences, University of Macau, Macao SAR 999078, China; 2 Patent Examination Cooperation Guangdong Center of the Patent Office, China National Intellectual Property Administration, Guangzhou 510555, China; 3 Department of Pharmaceutical sciences, Faculty of Health Sciences, University of Macau, Macao SAR 999078, China; 4 Zhuhai UM Science & Technology Research Institute, Zhuhai 519031, China

**Keywords:** *Ophiopogon japonicus*, *Liriope spicata* var. *prolifera*, HPLC-DAD-ELSD, anticancer, steroidal saponins, homoisoflavonoids

## Abstract

The tuberous root of *Ophiopogon japonicus* (Thunb.) Ker-Gawl. is a well-known Chinese medicine also called Maidong (MD) in Chinese. It could be divided into “Chuanmaidong” (CMD) and “Zhemaidong” (ZMD), according to the geographic origins. Meanwhile, the root of *Liriope spicata* (Thunb.) Lour. var. *prolifera* Y. T. Ma (SMD) is occasionally used as a substitute for MD in the market. In this study, a reliable pressurized liquid extraction and HPLC-DAD-ELSD method was developed for the simultaneous determination of nine chemical components, including four steroidal saponins (ophiopojaponin C, ophiopogonin D, liriopesides B and ophiopogonin D’), four homoisoflavonoids (methylophiopogonone A, methylophiopogonone B, methylophiopogonanone A and methylophiopogonanone B) and one sapogenin (ruscogenin) in CMD, ZMD and SMD. The method was validated in terms of linearity, sensitivity, precision, repeatability and accuracy, and then applied to the real samples from different origins. The results indicated that there were significant differences in the contents of the investigated compounds in CMD, ZMD and SMD. Ruscogenin was not detected in all the samples, and liriopesides B was only found in SMD samples. CMD contained higher ophiopogonin D and ophiopogonin D’, while the other compounds were more abundant in ZMD. Moreover, the anticancer effects of the herbal extracts and selected components against A2780 human ovarian cancer cells were also compared. CMD and ZMD showed similar cytotoxic effects, which were stronger than those of SMD. The effects of MD may be due to the significant anticancer potential of ophiopognin D’ and homoisoflavonoids. These results suggested that there were great differences in the chemical composition and pharmacological activity among CMD, ZMD and SMD; thus, their origins should be carefully considered in clinical application.

## 1. Introduction

*Ophiopogon japonicus* (Thunb.) Ker-Gawl., or Maidong (MD) in Chinese, has been used as a Chinese medicine to treat symptoms such as coughing, sore throat, insomnia and constipation since ancient China [1]. Modern pharmacological studies show that MD possess various activities such as anti-oxidation [2,3,4], anti-inflammation [4,5,6], anticancer [4,7], cardiovascular protection [8,9] and anti-diabetes [10,11,12]. Through years of studies, it is found that the bioactive components in MD mainly include steroidal saponins and homoisoflavonoids [13]. Steroidal saponins are classified into spirostanol saponins and furostanol saponins based on their aglycone. Among them, ruscogenin-type and diosgenin-type saponins are the most dominant saponins in MD [13]. Homoisoflavonoids are a unique subclass of flavonoids containing one additional carbon atom between B and C rings, which mainly exist in Asparagaceae and Fabaceae families [14]. The homoisoflavonoids of MD are classified into two groups on the basis of the saturation of the C2–C3 bond; one group contains a saturated C2–C3 bond, and the other group comprises a double bond at the same position [13].

In China, MD mainly comes from Sichuan and Zhejiang provinces, which are called “Chuanmaidiong” (CMD) or “Zhemaidong” (ZMD), accordingly [13]. Because of the different cultivated environment and growth years, it is believed that the quality of MD in the two places are different, which would influence their pharmacological activities and clinical efficacy. Previous studies demonstrated that ZMD contained higher contents of homoisoflavonoids than CMD, and exhibited better antioxidative, anti-inflammatory and immunomodulatory activities [2,4,15]; however, they showed distinctive composition of saponins and so exerted selective cytotoxic effects on different cell lines [4]. Meanwhile, the root of *Liriope spicata* (Thunb.) Lour. var. *prolifera* Y. T. Ma (SMD), mainly produced in Hubei province, is occasionally used as a substitute for MD in clinical practice due to their similar therapeutic effects. Compared with MD, SMD contained higher contents of steroidal saponins and lower contents of homoisoflavonoids [16,17]. Therefore, it is important to develop an effective quality control method for MD and SMD for their rational application. 

To date, a series of analytical methods including UV-Vis spectrophotometry [1], near infrared spectroscopy [18], TLC [19,20], HPLC-UV [21,22], HPLC-ELSD [23,24] and HPLC-MS [2,4,15,16,17,25] have been developed for the quality evaluation of MD and/or SMD. Although HPLC-MS has the advantages of high resolution and high sensitivity, the immense cost hinders its wide use in routine laboratories. HPLC-DAD-ELSD is an alternative approach for the simultaneous determination of steroidal saponins and homoisoflavonoids in MD and SMD, by which homoisoflavonoids could be easily detected by DAD based on their maximum absorption wavelength, and steroidal saponins that lack of chromophore could be detected by ELSD, a universal detector. 

In recent years, studies showed that MD exhibited an anticancer effect in various types of cancers. For example, the steroidal saponins and flavonoids of MD inhibited the proliferation of A549 cells [8]. Ophiopogonin D inhibited tumor growth by inducing apoptosis on non-small cell lung carcinoma mouse model [26]. Ophiopogonin D’ inhibited the proliferation of prostate cancer cells in vitro and in vivo [27,28]. However, the anticancer effects of different components in MD were rarely compared.

**Figure 1:**
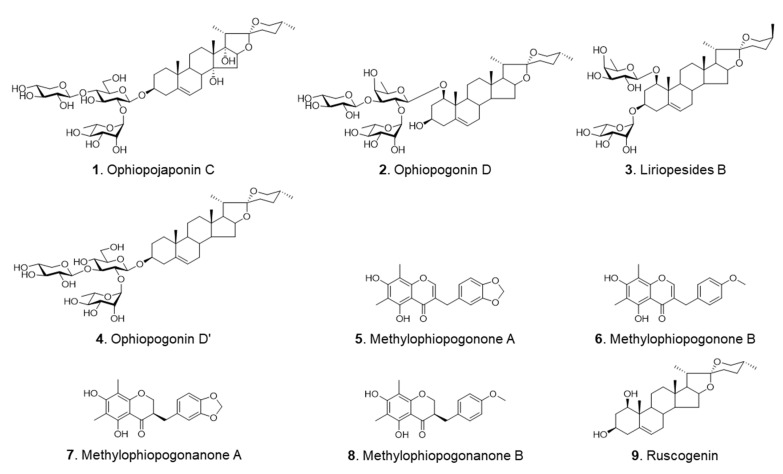
Chemical structures of the investigated compounds.

In the current study, a reliable pressurized liquid extraction (PLE) and HPLC-DAD-ELSD method was developed for the simultaneous determination of nine chemical components, including four steroidal saponins [ophiopojaponin C (**1**), ophiopogonin D (**2**), liriopesides B (**3**) and ophiopogonin D’ (**4**)], four homoisoflavonoids [methylophiopogonone A (**5**), methylophiopogonone B (**6**), methylophiopogonanone A (**7**) and methylophiopogonanone B (**8**)] and one sapogenin [ruscogenin (**9**)] (Figure 1) in CMD, ZMD and SMD. Furthermore, the cytotoxic effects of the herbal extracts and selected compounds against A2780 human ovarian cancer cells were also compared. 

## 2. Results and Discussion

### 2.1. Optimization of PLE Conditions

**Figure 2:**
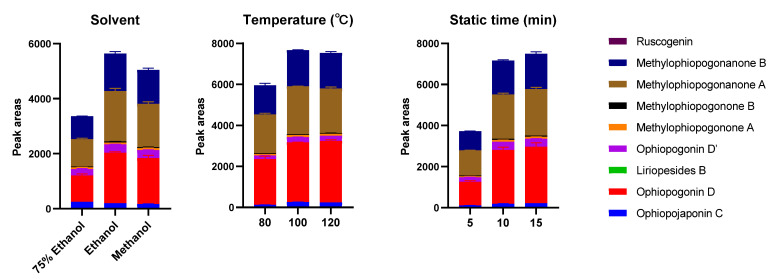
Influences of solvent, temperature, and static extraction time on PLE of the investigated compounds. To determine one of the parameters, the others were set at the definite values (solvent, ethanol; temperature, 100 °C; static extraction time, 10 min).

PLE is a modern and green technique which applies high temperature and high pressure to achieve quick, efficient, and repeatable sample extraction [29]; therefore, it was employed in this study to extract the investigated compounds from the herbal samples. To achieve the best extraction performance, the PLE procedure was optimized using the sample CMD-1, and the peak areas of the nine analytes were used as indicators to evaluate the extraction efficiency because all peaks’ areas varied at the same trend with the changes in parameters. The parameters, including the type of solvent (75% ethanol, absolute ethanol and methanol), temperature (80 °C, 100 °C and 120 °C), and static extraction time (5 min, 10 min and 15 min) were investigated by using a univariate approach, while other conditions were kept constant (pressure, 1500 psi; flush volume, 40% and extraction cycle, 1). As shown in Figure 2, absolute ethanol had the highest extraction efficiency. In addition, the peak areas would not increase with the rise in temperature and the extension of static extraction time when they reached 100 °C and 10 min, respectively. In addition, the recovery of the PLE was determined by performing consecutive extractions on the same sample under the optimized PLE conditions, until no investigated compounds were detected. The extraction recovery was calculated based on the total extracted amount of the investigated components during the consecutive extractions and the rate of the first-time extraction was nearly 100%. Considering the results of optimization and extraction recovery, the optimized PLE conditions were extraction solvent, absolute ethanol; temperature, 100 °C; static extraction time, 10 min; pressure, 1500 psi; flush volume, 40%; cycle, 1; and number of extraction times, 1.

### 2.2. Validation of the HPLC Method

The linearity, regression, and linear ranges of the nine analytes were summarized in Table 1. The results indicated good linearity (*R*^2^ > 0.9930) between the peak area and the concentration of the investigated compounds. The LODs and LOQs of saponins and sapogenin were under 26.00 μg/mL and 52.00 μg/mL, respectively; meanwhile, those of homoisoflavonoids were less than 0.08 μg/mL and 0.24 μg/mL, which suggested that DAD had better sensitivity than ELSD. The data of precision, repeatability and recovery were shown in Table 2. The intra-day and inter-day precision (RSD) of the analytes were between 0.17% and 0.95% and 0.40% and 3.93%, respectively. The repeatability expressed as RSD of the analytes at low, middle and high levels of the tested sample was less than 4.04%, 3.63% and 3.41%, respectively, except liriopesides B and ruscogenin, which were not detected in the CMD-1 sample. The recovery of the investigated compounds fell within the range between 94.4% and 105.3%. These results showed that the developed HPLC-DAD-ELSD method could simultaneously determine the analytes in MD and SMD with good sensitivity, precision, repeatability and accuracy within the designated range.

### 2.3. Quantification of the Investigated Compounds in CMD, ZMD and SMD 

**Figure 3:**
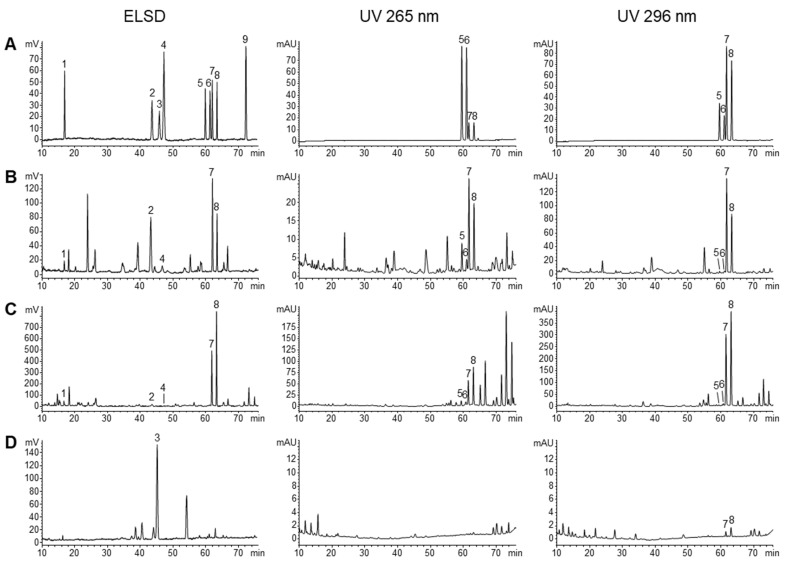
Typical HPLC chromatograms of mixed standards (**A**), CMD (**B**), ZMD (**C**) and SMD (**D**). The compounds numbers are the same as in Figure 1.

**Figure 4:**
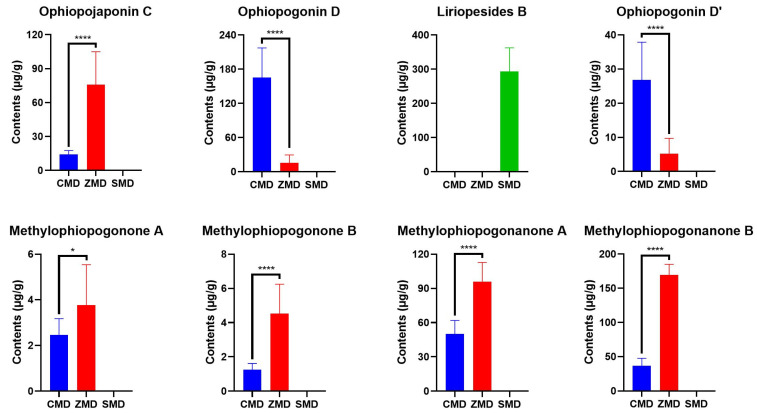
Comparison of the contents of the investigated compounds in CMD, ZMD and SMD (* *p* < 0.05, **** *p* < 0.0001).

The validated HPLC-DAD-ELSD method was applied to determine the nine investigated compounds in 14 batches of CMD, 8 batches of ZMD and 4 batches of SMD. Although all the analytes could be detected by ELSD, DAD was used to detect homoisoflavonoids due to its better sensitivity. Typical HPLC chromatograms of the mixed standards, CMD, ZMD and SMD were shown in Figure 3, and the contents of the investigated compounds were summarized in Table 3. The results showed that the amounts of the analytes varied greatly among CMD, ZMD and SMD (Figure 4), which may be derived from their different species, geographic origins and growth years.

In the Chinese Pharmacopoeia, the total saponins in MD are determined by UV spectrophotometry using ruscogenin as the reference standard and the saponins should be hydrolyzed to sapogenins by perchloric acid prior to the analysis [1]. In this study, ruscogenin was not detected in all the samples, which indicated that free ruscogenin hardly existed in MD and SMD. Among the investigated saponins, liriopesides B was only found in SMD, and it was also the only saponin detected in SMD, with a mean content of 293.08 μg/g. Ophiopogonin D and ophiopogonin D’ in CMD (165.08 μg/g and 26.83 μg/g) were significantly higher than in ZMD (25.28 μg/g and 8.39 μg/g), while the situation of ophiopojaponin C was contrary (14.29 μg/g in CMD and 75.93 μg/g in ZMD). It was noteworthy that ophiopogonin D was the quality control marker of MD in Hong Kong Chinese Materia Medica Standards, of which the content should not be less than 0.010%, i.e.,100 μg/g [30]. According to this requirement, all the ZMD samples were unqualified. Therefore, this marker may be unsuitable and further investigation should be conducted. In terms of homoisoflavonoids, all the four tested compounds were much higher in ZMD than in CMD, with the total amounts of 240.99–297.46 μg/g and 55.36–141.69 μg/g, respectively. In addition, they were hardly detected in SMD samples. The above results were consistent with previous reports [2,4,15,16,17,25].

### 2.4. Comparison of the Chemical Composition of CMD, ZMD and SMD by Multivariate Statistical Analysis

**Figure 5:**
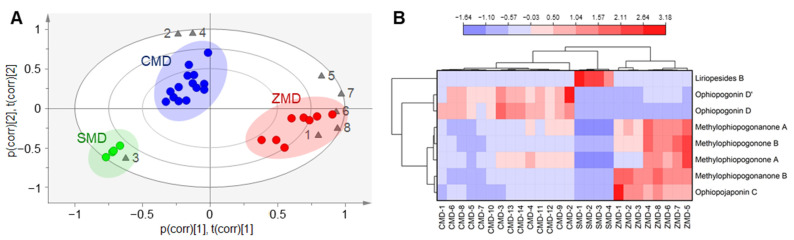
(**A**) PCA biplot of CMD (blue circles), ZMD (red circles) and SMD (green circles) based on the investigated compounds (gray triangles). The compound numbers are the same as in Figure 1. (**B**) Heatmap with HCA dendrogram of the tested samples and the investigated components.

Multivariate statistical analysis was applied to further compare the chemical differences among CMD, ZMD and SMD. Principal component analysis (PCA) is a common multivariate statistical analysis technique that reduces the dimensionality of datasets through orthogonal transformation to make the relationship between samples more intuitive [31]. PCA was carried out based on the contents of the investigated components. The cumulative values of R^2^X and Q^2^ were 0.979 and 0.814, respectively, indicating the good quality of the PCA model. A biplot that combined the score plot and loading plot was present in Figure 5A. The 26 samples could be clearly divided into three groups, namely CMD, ZMD and CMD, respectively. In addition, liriopesides B (**3**) was one of the main components of SMD; ophiopogonin D (**2**) and ophiopogonin D’ (**4**) were the most prominent in CMD; while ophiopojaponin C (**1**), methylophiopogonone A (**5**), methylophiopogonone B (**6**), methylophiopogonanone A (**7**) and methylophiopogonanone B (**8**) were more abundant in ZMD.

Hierarchical cluster analysis (HCA) is a statistical method for finding the hierarchy of clusters in the data based on specified characteristics. In this study, HCA and heatmap were used to visualize the differences among CMD, ZMD and SMD. After data standardization, HCA was performed using the method of group average and squared Euclidean distance as measurement. As shown in Figure 5B, CMD, ZMD and SMD were separated from each other and formed three clusters. In addition, the compounds were also divided into three clusters based on their contents in the sample, with liriopesides B which was only found in SMD in the first cluster, ophiopogonin D and ophiopogonin D’ which were higher in CMD in the second cluster, and the other five components which were more abundant in ZMD in the third cluster. Although CMD and SMD were from different species, they were closer in the HCA dendrogram, probably because they both contained lower levels of homoisoflavonoids and ophiopojaponin C.

The results of PCA and HCA were consistent, and both of them were in accordance with our quantification results. From the above results, the characteristic components of CMD, ZMD and SMD were clarified, and the samples from different origins could be discriminated based on the contents of these compounds. Therefore, multiple component determination would be a better strategy for quality control of MD and SMD.

### 2.5. In Vitro Anticancer Activity of CMD, ZMD and SMD Extracts and Their Components

**Figure 6:**
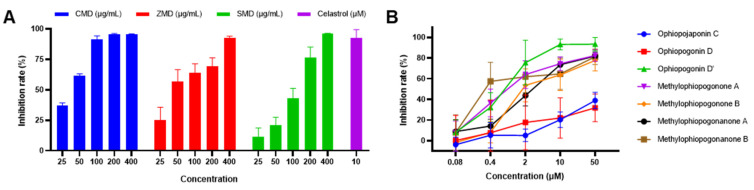
Inhibition effect of different herbal extracts (**A**) and the investigated compounds (**B**) on A2780 cells. Data presented as mean ± SD (n = 3).

In order to evaluate the in vitro anticancer activity of CMD, ZMD and SMD extracts and their components, MTT assay was used to examine their inhibitory effect on A2780 human ovarian cancer cells. Considering that the chemical profiles in MD and SMD from the same origin were similar, one sample from each group, namely CMD-1, ZMD-1 and SMD-1 were chosen as the representative for the activity evaluation. As shown in Figure 6 and Table 4, CMD, ZMD and SMD extracts inhibited the proliferation of A2780 cells in concentration-dependent manners. The IC_50_ values of CMD, ZMD and SMD were 0.967, 0.892 and 6.251 mg crude drug/mL, indicating that CMD and ZMD showed similar cytotoxic effects against A2780 cells, which were much stronger than SMD. Among the investigated components, ophiopogonin D’ exhibited the most prominent cytotoxic effect with IC_50_ value of 0.89 μM, followed by the four homoisoflavonoids (methylophiopogonone A, methylophiopogonone B, methylophiopogonanone A and methylophiopogonanone B), with IC_50_ values between 2.61 and 8.25 μM. The anticancer effects of the other compounds (ophiopojaponin C and ophiopogonin D) were weaker, with IC_50_ values over 50 μM.

These results suggested that ophiopogonin D’ and homoisoflavonoids may play important roles in the anticancer activity of MD. ZMD contained higher contents of homoisoflavonoids (273.61 μg/g) and showed the strongest cytotoxic potential. Although the content of homoisoflavonoids in CMD was relatively low (90.32 μg/g), it contained a certain amount of ophiopogonin D’ (26.83 μg/g), so CMD exhibited comparable anticancer activity to ZMD. SMD hardly contained ophiopogonin D’ and homoisoflavonoids, and showed a weaker cytotoxic effect compared with CMD and ZMD. Liriopesides B, the main component of SMD, was reported to suppress the proliferation of A2780 cells with IC_50_ value of 29.355 μM for 48 h [32], which may contribute to the anticancer activity of SMD. However, the anticancer ability of liriopesides B was not evaluated in the present study due to the limited amount of the reference standard. The anticancer effect of ruscogenin was not assessed either as it was not detected in all samples.

Actually, the anticancer activity of ophiopogonin D’ has been proven in previous reports [27,28]. It inhibited the proliferation of prostate cancer cells such as PC3 cells and LNCaP cells, but did not decrease the viability of human peripheral blood mononuclear cells. It also suppressed the growth of PC3 and DU145 xenograft tumors in BALB/c nude mice. The mechanism of action was considered to be related to the induction of receptor-interacting serine/threonine-protein kinase 1 (RIPK1)- and mixed lineage kinase domain-like protein (MLKL)-dependent necroptosis [27,28]. However, to the best of our knowledge, there were few studies on the anticancer effect and mechanism of homoisoflavonoids from MD and thus further investigation should be carried out in the future.

## 3. Materials and Methods

### 3.1. Materials and Reagents

Samples of *Ophiopogon japonicus* were collected from the Sichuan and Zhejiang provinces of China. Samples of *Liriope spicata* var. *prolifera* were collected from the Hubei province of China. The detailed sample information was listed in Table 5. The botanical origin of materials was identified by the corresponding author. The voucher specimens (CMD-1~CMD-14, ZMD-1~ZMD-8 and SMD-1~SMD-4) were deposited at the Institute of Chinese Medical Sciences, University of Macau, Macao SAR, China.

The reference standards of ophiopojaponin C, ophiopogonin D, liriopesides B, ophiopognin D’, methylophiopogonone A, methylophiopogonone B, methylophiopogonanone A, methylophiopogonanone B and ruscogenin were purchased from Baoji Herbest Bio-Tech Co., Ltd. (Baoji, China). Anhydrous ethanol was purchased from Fuyu Fine Chemical Co., Ltd. (Tianjin, China). HPLC grade acetonitrile was purchased from Merck (Darmstadt, Germany). A2780 human ovarian cancer cell line was purchased from KeyGEN BioTECH Ltd. (Jiangsu, China). DMEM medium, fetal bovine serum (FBS), penicillin and streptomycin were purchased from Gibco, Thermo Fisher Scientific (Waltham, MA, USA). Dimethyl sulfoxide (DMSO), 3-(4, 5-Dimethylthiazol-2-yl)-2, 5-diphenyl tetrazolium bromide (MTT) were purchased from Sigma (St. Louis, MO, USA). Deionized water was prepared by Millipore Milli Q-Plus system (Billerica, MA, USA).

### 3.2. Sample Preparation

The collected samples were dried in the oven at 40 °C overnight and pulverized into fine powders. Then, sample preparation was performed by using PLE on a Dionex ASE350 system (Dionex Corp., Sunnyvale, CA, USA). Dried powdered samples (1.00 g) were homogeneously mixed with the same amount of diatomaceous earth and placed into an 11 mL stainless steel extraction cell, and extracted under the optimized extraction conditions: solvent, absolute ethanol; temperature, 100 °C; pressure, 1500 psi; static extraction time, 10 min; flush volume, 40%; cycle, 1; number of extraction times, 1. The extract was then concentrated to dryness and re-dissolved with 1 mL methanol. After centrifugation (Eppendorf, Hamburg, Germany) at 13,000 rpm for 5 min, the supernatant was filtered through a 0.45 μm filter (Agilent Technologies, Santa Clara, CA, USA) before HPLC analysis. For the samples containing high contents of analytes, appropriate dilution was made to avoid the concentration beyond the linear range.

### 3.3. HPLC-DAD-ELSD Analysis

HPLC-DAD-ELSD analyses were performed on an Agilent 1200 HPLC system, equipped with an online vacuum degasser, a quaternary pump, an autosampler, a column oven, a diode-array detector, an evaporative light scattering detector and controlled by Agilent ChemStation. An Agilent Zorbax SB-C_18_ (250 mm × 4.6 mm, 5 μm) column with a Zorbax SB-C_18_ (12.5 mm × 4.6 mm, 5 μm) guard column was used for separation. The gradient elution with a mobile phase constituted with water (A) and acetonitrile (B) was used as follows: 0–20 min, 30–40% B; 20–44 min, 40–43% B; 44–71 min, 43–70% B; 71–76 min, 70–100% B. The flow rate was 1.0 mL/min, column temperature was 25 °C and injection volume was 10 μL. The homoisoflavonoids were detected by DAD, and the detection wavelengths were set at 265 nm for methylophiopogonone A and methylophiopogonone B, and 296 nm for methylophiopogonanone A and methylophiopogonanone B. ELSD was used to determine ophiopojaponin C, ophiopogonin D, liriopesides B, ophiopogonin D’ and ruscogenin, and the conditions were drift tube temperature, 60 °C; nebulizing gas (N_2_) flow rate, 1.5 mL/min; gain, 16.

### 3.4. Validation of the HPLC Method

#### 3.4.1. Calibration Curves, LOD and LOQ

Mixed standards stock solutions containing the nine reference compounds were prepared in methanol and diluted in series. At least five concentrations of the solution were analyzed by the developed method in duplicates, and the calibration curves were constructed by plotting the peak areas versus the concentration of each analyte. LOD and LOQ of each analyte was determined at a signal to noise ratio (S/N) of about 3 and 10, respectively.

#### 3.4.2. Precision and Repeatability

The precision of the developed method was determined by intra- and inter-day variation. For intra-day variation, the mixed standards solution was analyzed 6 times within the same day; while, for inter-day variation, the solution was examined in duplicates for three consecutive days. The RSD of the peak area of each analyte was used to assess the precision of the method.

The repeatability was assessed at three levels (0.80 g, 1.00 g and 1.20 g) of the sample CMD-1 (n = 3 for each concentration). The samples were extracted and analyzed by the developed method. The repeatability of the method was evaluated by calculating the RSD of the peak area of each analyte.

#### 3.4.3. Recovery

The recovery was used to evaluate the accuracy of the method. Known amounts of individual standards were added into a certain amount (0.50 g) of sample CMD-1 (n = 3). The samples were extracted and analyzed by the developed method and the recovery was calculated in percentage using the following equation: recovery (%) = (detected amount − original amount)/spiked amount × 100%.

### 3.5. Cell Culture

A2780 ovarian cancer cells were cultivated in DMEM medium containing 10% FBS, 100 μg/mL penicillin/streptomycin and maintained in a humidified incubator at 37 °C with 5% CO_2_. The medium was changed twice a day and the cells used in the experiment were at an exponential phase.

### 3.6. Cytotoxicity Assay

The cytotoxic activity was evaluated by the MTT method. The herbal extracts and selected standards were dissolved in DMSO at the concentrations of 400 mg/mL and 50 mM, respectively. Then, the solutions were diluted in culture medium to obtain gradient concentrations. Celastrol (10 μM) served as the positive control. A2780 cells (7 × 10^4^ cells/mL) were plated into 96-well plates at 100 μL/well. After 18 h of incubation, the medium was replaced by different concentrations of extracts or compounds. After incubation for 48 h, 100 μL of MTT (1 mg/mL) was added into each well and incubated for 4 h. Then the culture medium was removed and 100 μL of DMSO was added to dissolve the formazan crystals. The absorbance of the resulting solution was recorded at 570 nm using a microplate reader (Perkin Elmer, Singapore City, Singapore). Three independent experiments were performed in triplicate.

### 3.7. Data Analysis

Data from replicate experiments were expressed as the mean ± SD and were analyzed using the GraphPad Prism 9.0 (GraphPad Software Inc., San Diego, CA, USA). Analysis of variance and student’s *t*-test were used to evaluate the significance. A value of *p* < 0.05 was considered statistically significant. Heatmap and clustering analysis was performed using OriginPro 2022 (OriginLab, Northampton, MA, USA). PCA was carried out by SIMCA 14.1 (Umetrics, Umeå, Sweden).

## 4. Conclusions

In this study, a reliable PLE and HPLC-DAD-ELSD method was developed to determine the steroidal saponins, homoisoflavonoids and sapogenin in MD samples produced in Sichuan and Zhejiang, as well as SMD samples produced in Hubei. The developed method is an easy and economic method for quality control of MD and SMD and discriminates MD and SMD from different origins. From the data in our research, there were great differences among ZMD, CMD and SMD in their contents of saponins and homoisoflavonoids. In terms of anticancer activity, CMD and ZMD showed comparable cytotoxic effects, which may be mainly because of the significant anticancer effects of ophiopogonin D’ and homoisoflavonoids. Since there were great differences in the chemical composition and anticancer effect among ZMD, CMD and SMD, the origins of MD and SMD should be carefully considered in practical applications.

## Figures and Tables

**Table 1:** Linear regression data, LOD, LOQ of the investigated compounds.

Analyte	Calibration Curve			LOD (μg/mL)	LOQ (μg/mL)
	Regressive Equation	Test Range (μg/mL)	R^2^		
Ophiopojaponin C	y = 12.53x − 141.06	24.20–121.10	0.9992	12.10	24.20
Ophiopogonin D	y = 16.71x − 333.27	50.70–182.00	0.9998	25.30	50.70
Liriopesides B	y = 18.02x − 630.93	52.00–182.00	0.9982	26.00	52.00
Ophiopogonin D’	y = 18.99x − 597.45	49.00–247.00	0.9930	24.90	49.00
Methylophiopogonone A	y = 39.79x − 64.29	11.90–59.40	1.0000	0.08	0.24
Methylophiopogonone B	y = 40.59x − 55.02	11.20–56.10	0.9999	0.08	0.23
Methylophiopogonanone A	y = 36.77x − 58.73	12.30–61.70	0.9999	0.08	0.23
Methylophiopogonanone B	y = 32.27x − 51.54	11.90–59.40	1.0000	0.07	0.22
Ruscogenin	y = 24.294x − 438.83	24.90–124.00	0.9987	12.40	24.90

**Table 2:** Precision, Repeatability and Accuracy of the Investigated Compounds.

Analyte	Precision		Repeatability			Accuracy	
	Intra-Day RSD, % (n = 6)	Inter-Day RSD, % (n = 6)	Low RSD, % (n = 3)	Medium RSD, % (n = 3)	High RSD, % (n = 3)	Recovery, %	RSD, % (n = 3)
Ophiopojaponin C	0.17	1.45	0.67	1.37	3.41	103.9	0.35
Ophiopogonin D	0.43	0.60	3.56	2.10	2.37	105.3	0.68
Liriopesides B	0.28	1.64	- *	-	-	99.7	0.63
Ophiopogonin D’	0.95	0.70	4.04	1.48	0.63	102.0	0.45
Methylophiopogonone A	0.27	0.40	3.67	1.01	1.77	94.4	0.21
Methylophiopogonone B	0.29	0.88	2.82	2.19	3.26	99.2	0.88
Methylophiopogonanone A	0.30	1.22	4.01	0.95	0.33	96.5	0.40
Methylophiopogonanone B	0.30	1.23	2.96	3.63	3.17	95.0	0.22
Ruscogenin	0.44	3.93	-	-	-	96.1	4.21

* Not detected.

**Table 3:** Contents (μg/g) of the investigated compounds in CMD, ZMD and SMD.

Samples	1 ^1^	2	3	4	5	6	7	8	9	TS ^2^	TF ^3^
CMD-1	11.26	164.12	- ^4^	14.64	1.58	0.97	36.92	34.45	-	190.02	73.92
CMD-2	20.01	212.38	-	60.15	3.20	1.79	73.69	63.01	-	292.54	141.69
CMD-3	11.35	266.85	-	34.72	3.16	1.60	47.08	41.11	-	312.92	92.95
CMD-4	20.06	183.02	-	22.58	3.36	1.58	60.49	42.75	-	225.66	108.18
CMD-5	10.92	98.37	-	19.75	1.27	0.49	35.29	18.31	-	129.04	55.36
CMD-6	13.10	162.45	-	25.95	2.33	1.19	36.17	27.23	-	201.50	66.92
CMD-7	11.15	88.41	-	18.96	1.96	0.81	42.94	26.71	-	118.52	72.42
CMD-8	16.64	154.7	-	24.23	1.58	0.93	33.06	22.85	-	195.57	58.42
CMD-9	16.17	158.19	-	30.96	2.44	1.41	56.17	40.61	-	205.32	100.63
CMD-10	9.07	99.10	-	17.95	2.02	1.09	51.81	38.81	-	126.12	93.73
CMD-11	14.66	154.16	-	23.54	2.91	1.48	54.10	40.24	-	192.36	98.73
CMD-12	14.88	127.67	-	23.36	3.25	1.46	63.14	41.93	-	165.91	109.78
CMD-13	13.83	224.59	-	29.10	2.52	1.26	54.95	36.89	-	267.52	95.62
CMD-14	16.93	217.14	-	29.79	3.00	1.49	53.21	38.41	-	263.86	96.11
Mean	14.29	165.08	/	26.83	2.47	1.25	49.93	36.67	/	206.20	90.32
ZMD-1	140.92	-	-	-	1.65	2.69	72.46	181.12	-	140.92	257.92
ZMD-2	84.08	-	-	-	1.95	2.70	87.64	194.18	-	84.08	286.47
ZMD-3	85.11	-	-	-	1.80	2.48	73.29	163.42	-	85.11	240.99
ZMD-4	63.64	33.37	-	8.73	4.92	5.72	114.82	171.26	-	105.74	296.72
ZMD-5	53.97	21.13	-	6.45	6.45	6.96	113.95	163.82	-	81.55	291.18
ZMD-6	52.61	23.68	-	8.90	4.09	5.09	100.59	144.14	-	85.19	253.91
ZMD-7	59.69	27.45	-	10.07	4.56	5.90	96.42	157.31	-	97.21	264.19
ZMD-8	67.45	20.77	-	7.79	4.73	4.71	108.17	179.85	-	96.01	297.46
Mean	75.93	25.28	/	8.39	3.77	4.53	95.92	169.39	/	96.98	273.61
SMD-1	-	-	377.44	-	+ ^5^	+	+	+	-	377.44	/
SMD-2	-	-	301.65	-	+	+	+	+	-	301.65	/
SMD-3	-	-	284.67	-	-	-	+	+	-	284.67	/
SMD-4	-	-	208.55	-	-	-	+	+	-	208.55	/
Mean	/	/	293.08	/	/	/	/	/	/	293.08	/

^1^ The compounds numbers are the same as in Figure 1. ^2^ Total saponins: total contents of ophiopojaponin C, ophiopogonin D, liriopesides B and ophiopognin D’ (compound **1**–**4**). ^3^ Total flavonoids: total contents of methylophiopogonone A, methylophiopogonone B, methylophiopogonanone A and methylophiopogonanone B (compound **5–8**). ^4^ Not detected. ^5^ Under the limit of quantitation.

**Table 4:** IC_50_ of different herbal extracts and the investigated compounds against A2780 cells.

Analyte	IC_50_ (Mean ± SD, n = 3)
CMD extract	35.8 ± 0.5 μg/mL (0.967 ± 0.012 mg crude drug/mL)
ZMD extract	64.5 ± 17.7 μg/mL (0.892 ± 0.245 mg crude drug/mL)
SMD extract	119.9 ± 25.6 μg/mL (6.251 ± 1.335 mg crude drug/mL)
Ophiopojaponin C	>50 μM
Ophiopogonin D	>50 μM
Ophiopogonin D’	0.89 ± 0.64 μM
Methylophiopogonone A	2.61 ± 2.07 μM
Methylophiopogonone B	8.25 ± 3.31 μM
Methylophiopogonanone A	3.98 ± 2.38 μM
Methylophiopogonanone B	3.25 ± 3.46 μM

**Table 5:** Information of *Ophiopogon japonicus* and *Liriope spicata* var. *prolifera* samples.

No.	Code	Species	Origin
1	CMD-1	*Ophiopogon japonicus*	Laoma, Santai, Sichuan, China
2	CMD-2	*Ophiopogon japonicus*	Laoma, Santai, Sichuan, China
3	CMD-3	*Ophiopogon japonicus*	Laoma, Santai, Sichuan, China
4	CMD-4	*Ophiopogon japonicus*	Laoma, Santai, Sichuan, China
5	CMD-5	*Ophiopogon japonicus*	Laoma, Santai, Sichuan, China
6	CMD-6	*Ophiopogon japonicus*	Laoma, Santai, Sichuan, China
7	CMD-7	*Ophiopogon japonicus*	Xinde, Santai, Sichuan, China
8	CMD-8	*Ophiopogon japonicus*	Zhengsheng, Santai, Sichuan, China
9	CMD-9	*Ophiopogon japonicus*	Huanyuan, Santai, Sichuan, China
10	CMD-10	*Ophiopogon japonicus*	Laoma, Santai, Sichuan, China
11	CMD-11	*Ophiopogon japonicus*	Xinde, Santai, Sichuan, China
12	CMD-12	*Ophiopogon japonicus*	Laoma, Santai, Sichuan, China
13	CMD-13	*Ophiopogon japonicus*	Guangming, Santai, Sichuan, China
14	CMD-14	*Ophiopogon japonicus*	Lingxing, Santai, Sichuan, China
15	ZMD-1	*Ophiopogon japonicus*	Fuhai, Cixi, Zhejiang, China
16	ZMD-2	*Ophiopogon japonicus*	Fuhai, Cixi, Zhejiang, China
17	ZMD-3	*Ophiopogon japonicus*	Fuhai, Cixi, Zhejiang, China
18	ZMD-4	*Ophiopogon japonicus*	Shengshan, Cixi, Zhejiang, China
19	ZMD-5	*Ophiopogon japonicus*	Shengshan, Cixi, Zhejiang, China
20	ZMD-6	*Ophiopogon japonicus*	Shengshan, Cixi, Zhejiang, China
21	ZMD-7	*Ophiopogon japonicus*	Shengshan, Cixi, Zhejiang, China
22	ZMD-8	*Ophiopogon japonicus*	Shengshan, Cixi, Zhejiang, China
23	SMD-1	*Liriope spicata* var. *prolifera*	Hubei, China
24	SMD-2	*Liriope spicata* var. *prolifera*	Hubei, China
25	SMD-3	*Liriope spicata* var. *prolifera*	Hubei, China
26	SMD-4	*Liriope spicata* var. *prolifera*	Hubei, China

## Data Availability

The data presented in this study are contained within the article.

## References

[1] Chinese Pharmacopoeia Commission (2020). Ophiopogonis Radix. Chinese Pharmacopoeia of the People’s Republic of China, 2020 ed..

[2] Lin Y., Zhu D., Qi J., Qin M., Yu B. (2010). Characterization of homoisoflavonoids in different cultivation regions of *Ophiopogon japonicus* and related antioxidant activity. J. Pharm. Biomed. Anal..

[3] Xiong S.L., Li A., Huang N., Lu F., Hou D. (2011). Antioxidant and immunoregulatory activity of different polysaccharide fractions from tuber of *Ophiopogon japonicus*. Carbohydr. Polym..

[4] Zhao M., Xu W.-F., Shen H.-Y., Shen P.-Q., Zhang J., Wang D.-D., Xu H., Wang H., Yan T.-T., Wang L. (2017). Comparison of bioactive components and pharmacological activities of Ophiopogon japonicas extracts from different geographical origins. J. Pharm. Biomed. Anal..

[5] Kou J., Sun Y., Lin Y., Cheng Z., Zheng W., Yu B., Xu Q. (2005). Anti-inflammatory activities of aqueous extract from Radix *ophiopogon japonicus* and its two constituents. Biol. Pharm. Bull..

[6] Kitahiro Y., Koike A., Sonoki A., Muto M., Ozaki K., Shibano M. (2018). Anti-inflammatory activities of Ophiopogonis Radix on hydrogen peroxide-induced cellular senescence of normal human dermal fibroblasts. J. Nat. Med..

[7] Chen J., Yuan J., Zhou L., Zhu M., Shi Z., Song J., Xu Q., Yin G., Lv Y., Luo Y. (2017). Regulation of different components from *Ophiopogon japonicus* on autophagy in human lung adenocarcinoma A549 Cells through PI3K/Akt/mTOR signaling pathway. Biomed. Pharmacother..

[8] Kou J., Yu B., Xu Q. (2005). Inhibitory effects of ethanol extract from Radix *Ophiopogon japonicus* on venous thrombosis linked with its endothelium-protective and anti-adhesive activities. Vasc. Pharmacol..

[9] Cai H.-Z., Tang Y.-P., Chen X.-Y., Xie H.-B., Chen Q.-Y., Xu Z.-L. (2019). Ophiopogon japonicas (Linn. f.) Ker-Gawl. extract ameliorates chronic heart failure in rats. Trop. J. Pharm. Res..

[10] Ding L., Li P., Lau C.B.S., Chan Y.W., Xu D., Fung K.-P., Su W. (2012). Mechanistic Studies on the Antidiabetic Activity of a Polysaccharide-rich Extract of Radix Ophiopogonis. Phytother. Res..

[11] Li P.-B., Lin W.-L., Wang Y.-G., Peng W., Cai X.-Y., Su W.-W. (2012). Antidiabetic activities of oligosaccharides of Ophiopogonis japonicus in experimental type 2 diabetic rats. Int. J. Biol. Macromol..

[12] Wang H.-Y., Guo L.-X., Hu W.-H., Peng Z.-T., Wang C., Chen Z.-C., Liu E.Y.L., Dong T.T.X., Wang T.-J., Tsim K.W.K. (2019). Polysaccharide from tuberous roots of *Ophiopogon japonicus* regulates gut microbiota and its metabolites during alleviation of high-fat diet-induced type-2 diabetes in mice. J. Funct. Foods..

[13] Chen M.-H., Chen X.-J., Wang M., Lin L.-G., Wang Y.-T. (2016). *Ophiopogon japonicus*–A phytochemical, ethnomedicinal and pharmacological review. J. Ethnopharmacol..

[14] Lin L.-G., Liu Q.-Y., Ye Y. (2014). Naturally occurring homoisoflavonoids and their pharmacological activities. Planta Med..

[15] Lu X., Tong W., Wang S., Li J., Zheng J., Fan X., Liu L. (2017). Comparison of the chemical consituents and immunomodulatory activity of ophiopogonis radix from two different producing areas. J. Pharm. Biomed. Anal..

[16] Wu Y., Dong Z., Wu H., Ding W., Zhao M., Shi Q., Wang Q. (2014). Comparative studies on Ophiopogonis and Liriopes based on the determination of 11 bioactive components using LC-MS/MS and Hierarchical clustering analysis. Food Res. Int..

[17] Luo H., Ming L.S., Tong T.T., Tang Y., Yang J., Shen L., Cui H., Yang A., Huang H. (2020). Chemical Comparison of Ophiopogonis radix and Liriopes radix Based on Quantitative Analysis of Multiple Components by HPLC Coupled with Electrospray Ionization Tandem Triple Quadrupole Mass Spectrometry. J. AOAC Int..

[18] Dai S., Lin Z., Xu B., Wang Y., Shi X., Qiao Y., Zhang J. (2018). Metabolomics data fusion between near infrared spectroscopy and high-resolution mass spectrometry: A synergetic approach to boost performance or induce confusion. Talanta.

[19] Ge Y., Chen X., Godjevac D., Bueno P.C.P., Salome Abarca L.F., Jang Y.P., Wang M., Choi Y.H. (2019). Metabolic Profiling of Saponin-Rich *Ophiopogon japonicus* Roots Based on ^1^H NMR and HPTLC Platforms. Planta Med..

[20] Hua X., Hong H.-J., Zhang D.-Y., Liu Q., Leong F., Yang Q., Hu Y.-J., Chen X.-J. (2022). Rapid Screening of Lipase Inhibitors from Ophiopogonis Radix Using High-Performance Thin Layer Chromatography by Two Step Gradient Elution Combined with Bioautographic Method. Molecules.

[21] Ye G., Ye M., Guo D., Huang C. (2005). Determination of homoisoflavonoids in *Ophiopogon japonicus* by RP-HPLC. Chromatographia.

[22] Liu J., Chen X., Yang W., Zhang S., Wang F., Tang Z. (2010). Chemical Fingerprinting of Wild Germplasm Resource of *Ophiopogon japonicus* from Sichuan Basin, China by RP-HPLC Coupled with Hierarchical Cluster Analysis. Anal. Lett..

[23] Li X.-E., Wang Y.-X., Sun P., Liao D.-Q. (2016). Determination of saponin content in hang maidong and chuan maidong via HPLC-ELSD analysis. J. Anal. Methods Chem..

[24] Liu C.-H., Li M., Feng Y.-Q., Hu Y.-J., Yu B.-Y., Qi J. (2016). Determination of ruscogenin in ophiopogonis radix by high-performance liquid chromatography-evaporative light scattering detector coupled with hierarchical clustering analysis. Pharmacogn. Mag..

[25] Lyu C.-G., Kang C.-Z., Kang L.-P., Yang J., Wang S., He Y.-L., Deng A.-P., Wang H.-Y., Huang L.-Q., Guo L.-P. (2020). Structural characterization and discrimination of Ophiopogon japonicas (Liliaceae) from different geographical origins based on metabolite profiling analysis. J. Pharm. Biomed. Anal..

[26] Lee J.H., Kim C., Lee S.-G., Sethi G., Ahn K.S. (2018). Ophiopogonin D, a steroidal glycoside abrogates STAT3 signaling cascade and exhibits anti-cancer activity by causing GSH/GSSG imbalance in lung carcinoma. Cancers.

[27] Lu Z., Wang H., Zhu M., Song W., Wang J., Wu C., Kong Y., Guo J., Li N., Liu J. (2018). Ophiopogonin D’, a natural product from Radix ophiopogonis, induces in vitro and in vivo RIPK1-dependent and caspase-independent apoptotic death in androgen-independent human prostate cancer cells. Front. Pharmacol..

[28] Lu Z., Wu C., Zhu M., Song W., Wang H., Wang J., Guo J., Li N., Liu J., Li Y. (2020). Ophiopogonin D’ induces RIPK1-dependent necroptosis in androgen-dependent LNCaP prostate cancer cells. Int. J. Oncol..

[29] Mustafa A., Turner C. (2011). Pressurized liquid extraction as a green approach in food and herbal plants extraction: A review. Anal. Chim. Acta.

[30] Department of Health, Hong Kong Special Administrative Region, the People’s Republic of China (2010). Radix Ophiopogonis. Hong Kong Chinese Materis Medica Standards.

[31] Jolliffe I.T., Cadima J. (2016). Principal component analysis: A review and recent developments. Philos. Trans. A Math. Phys. Eng. Sci..

[32] Wang H., Yu H., Sun Y., Zhao H., Guo Z., Yu B. (2017). Liriopesides B inhibited cell growth and decreased CA125 level in human ovarian cancer A2780 cells. Nat. Prod. Res..

